# Acetaminophen Overdose in an Extremely Low-Birth-Weight Premature Infant: A Case Report and Review of the Literature

**DOI:** 10.7759/cureus.110671

**Published:** 2026-06-11

**Authors:** Amy Pham, Kirsten Ohler, Renee Petzel Gimbar, Trevonne M Thompson, De-Ann Pillers, Aarti Raghavan

**Affiliations:** 1 Pediatrics and Neonatology, University of Illinois Chicago, Chicago, USA; 2 Pharmacy, University of Illinois Chicago, Chicago, USA; 3 Emergency Medicine, University of Illinois Chicago, Chicago, USA

**Keywords:** acetaminophen toxicity, medication error, n-acetylcysteine, paracetamol overdose, premature infant

## Abstract

Acetaminophen (paracetamol, APAP) overdose is well characterized in pediatric and adult populations; however, reports of toxicity in premature neonates remain exceedingly rare. Existing literature describing acetaminophen overdose in preterm infants is limited to only four published cases. Acetaminophen metabolism in extremely premature neonates differs substantially from that of older children and adults because of developmental immaturity of glucuronidation and cytochrome P450 oxidative pathways, greater reliance on sulfation, and relatively preserved glutathione stores. These developmental differences may alter susceptibility to hepatotoxicity and complicate the interpretation of conventional toxicity thresholds and treatment strategies in this population.

This case highlights one of the smallest and youngest reported premature infants, born at 24 + 4 weeks gestation and weighing 640 g at the time of overdose, who survived a 10-fold intravenous (IV) acetaminophen overdose without apparent sequelae following treatment with N-acetylcysteine (NAC). The infant inadvertently received two supratherapeutic intravenous acetaminophen doses because of a medication error involving a non-formulary medication entry within the electronic medical record. Laboratory evaluation demonstrated elevated acetaminophen concentrations and transient coagulation abnormalities without evidence of hepatocellular injury. N-acetylcysteine therapy was initiated with subsequent normalization of coagulation studies and decline of acetaminophen concentrations to undetectable levels.

This report expands the limited literature on acetaminophen toxicity in premature neonates and emphasizes the growing importance of understanding neonatal pharmacokinetics as acetaminophen use in neonatal intensive care units increases, particularly for treatment of hemodynamically significant patent ductus arteriosus (PDA). Further investigation is needed to establish population-specific toxicity thresholds and evidence-based management strategies for extremely preterm infants.

## Introduction

Acetaminophen (APAP) is one of the most commonly used analgesic and antipyretic medications in neonates, with growing use in the treatment of hemodynamically significant patent ductus arteriosus (PDA), a persistent fetal vascular connection that can contribute to respiratory compromise and systemic hypoperfusion in premature infants. Compared with traditional cyclooxygenase inhibitors such as indomethacin and ibuprofen, acetaminophen may offer a more favorable side effect profile with less risk of renal dysfunction, gastrointestinal complications, and platelet inhibition, while also providing a non-surgical treatment option for medically unstable, extremely premature infants with very low birth weight. While its safety profile is well established in pediatric and adult populations, data regarding overdose in neonates, particularly premature infants, remain scarce. Neonates exhibit distinct metabolic pathways due to immature hepatic enzymatic systems, including reduced cytochrome P450 oxidative metabolism responsible for the production of the toxic metabolite N-acetyl-p-benzoquinone imine (NAPQI), potentially altering the pharmacokinetics and toxicity thresholds of acetaminophen. The existing literature on neonatal overdose is limited, with only four previously reported cases in premature infants. In this paper, we present a case of a profoundly premature infant born at 24 + 4 weeks of gestation with a birth weight of 590 g, representing one of the youngest gestational ages and lowest-weight premature infants reported in the literature to receive an intravenous (IV) acetaminophen overdose, and discuss the unique metabolic considerations that contribute to drug toxicity in this population.

Two physician verbal parental consent for publication was obtained; however, written consent could not be completed because the infant had been discharged and the case report was prepared four years later, at which time the mother was unable to return to the hospital to sign the documentation.

## Case presentation

**Figure 1:**
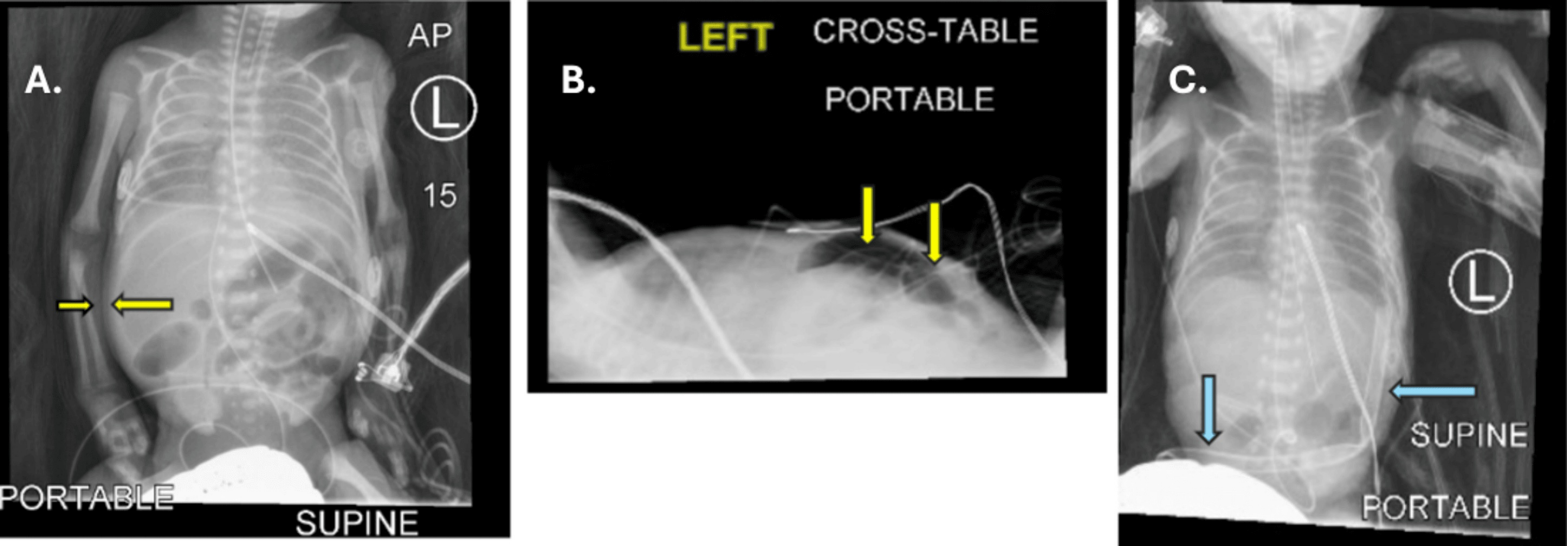
Radiographic findings of spontaneous intestinal perforation in an extremely premature neonate A. Supine abdominal radiograph demonstrating lucency concerning for free intraperitoneal air (yellow arrows). B. Cross-table lateral radiograph confirming pneumoperitoneum beneath the anterior abdominal wall (yellow arrows). C. Follow-up supine radiograph status post Penrose drain placement (blue arrows).

A Hispanic male infant born at 24 + 4 weeks gestational age, with a birth weight of 590 g, a history of respiratory distress syndrome, treated presumed sepsis, and grade 4 bilateral intraventricular hemorrhage, presented on day of life 12, with a current weight of 640 g, with worsened clinical status. An abdominal radiograph was obtained, demonstrating pneumoperitoneum (Figure 1). Radiographic findings of abdominal free air (Figure 1, yellow arrows) led to escalation of respiratory support to high-frequency oscillatory ventilation, development of hypotension requiring vasopressor support, and placement of a Penrose drain for spontaneous intestinal perforation (Figure 1, blue arrows). Fentanyl infusion was used for pain management during and after the procedure, which was subsequently transitioned to non-formulary intravenous acetaminophen on postoperative day 4.

On day of life 16, at a weight of 640 g, a medication error resulted in 100 mg/kg intravenous acetaminophen (non-formulary) every 8 hours being inadvertently ordered instead of 10 mg/kg intravenous acetaminophen and administered twice prior to identification of the overdose. The dosing error was recognized by the bedside nurse before the administration of a potential third dose. Once the medication overdose error was identified, the medication was discontinued, and management was guided by a multidisciplinary collaboration with a neonatologist, gastroenterologist, toxicologist, and clinical pharmacist. Laboratory data showed isolated coagulopathy without transaminitis and an elevated acetaminophen (APAP) level (Table 1). Based on literature review and recommendations from a toxicology specialist, N-acetylcysteine (NAC) was given 18 hours after the last dose of acetaminophen. The serum acetaminophen concentration declined to <10 mcg/mL 28 hours after the last dose of acetaminophen (10 hours after NAC). NAC was discontinued.

**Table 1:** Timeline of laboratory findings at baseline, 15 hours after the second acetaminophen dose, and 25 hours after the second acetaminophen dose ^A^Baseline laboratory values ^B^Laboratory values 24 hours after the first dose of 100 mg/kg IV acetaminophen and 15 hours after the second dose of 100 mg/kg IV acetaminophen ^C^Laboratory values 34 hours after the first dose of 100 mg/kg IV acetaminophen and 25 hours after the second dose of 100 mg/kg IV acetaminophen ALT: alanine transaminase, AST: aspartate aminotransferase, PT: prothrombin time, aPTT: activated partial thromboplastin time, INR: international normalized ratio, APAP: acetaminophen

Parameters	Values^A^	Values^B^ 15 hours after second acetaminophen dose	Values^C^ 25 hours after second acetaminophen dose	Reference values
Total bilirubin	3.4 mg/dL	4.2 mg/dL	4.1 mg/dL	0.0-1.2 mg/dL
Direct bilirubin	0.7 mg/dL	0.7 mg/dL	0.7 mg/dL	0.0-0.2 mg/dL
Alkaline phosphatase	620 μ/L	243 μ/L	188 μ/L	140-320 μ/L
ALT	6 μ/L	9 μ/L	12 μ/L	5-45 μ/L
AST	48 μ/L	34 μ/L	29 μ/L	20-60 μ/L
Protein	2.9 g/dL	3.8 g/dL	3.6 g/dL	4.0-8.0 g/dL
Albumin	4.2 g/dL	2.5 g/dL	2.4 g/dL	2.5-4.0 g/dL
PT	-	35 seconds	-	11.5-14.5 seconds
aPTT	-	47 seconds	-	25-36 seconds
INR	-	3.5	-	0.9-1.2
APAP level	-	29.6 mg/L	<10 mg/L	10-20 mg/L

Following Penrose drain placement for spontaneous intestinal perforation, the infant demonstrated rapid clinical improvement with stabilization of respiratory and hemodynamic status. Notably, before and after recognition of the acetaminophen overdose, the infant remained clinically stable without overt signs of hepatic dysfunction or bleeding, including absence of hepatomegaly, mucosal bleeding, or worsening intracranial hemorrhage despite the transient coagulation abnormalities observed on laboratory evaluation.

The infant remained clinically stable, completed his neonatal intensive care unit (NICU) course, and was discharged home. He was followed through two years of age in the NICU follow-up clinic, with no long-term gastrointestinal or coagulation abnormalities, although he required ventriculoperitoneal shunt placement for post-hemorrhagic hydrocephalus.

Review of literature was facilitated by the University of Illinois Chicago librarian through search engines including PubMed, Embase, CINAHL, and Web of Science (search phrases are noted in the Appendices). Case reports were included if they described an overdose in premature infants (PO, NG, or IV) until 40 weeks corrected postmenstrual age. Otherwise, all other cases were excluded, including those articles that were not written in English. A summary of the four case reports noted is presented in Table 2.

**Table 2:** Summary of published case reports of acetaminophen overdose in preterm infants compared with the case presentation APAP: acetaminophen, GA: gestational age, PMA: postmenstrual age, LFT: liver function test, INR: international normalized ratio, PT: prothrombin time, PTT: partial thromboplastin time

Article title	Author	Patient information	Overdose (mL/kg)	APAP level	Abnormal laboratory results after overdose were noted	Treatment
Our case	Pham et al.	GA at birth: 24 + 4 weeks, weight: 0.640 kg, PMA at time of overdose: 26 + 6	100 mg/kg IV q 8 hours (×2)	29.6 mg/L 25 hours after last dose	No increase in LFT; increase in INR (3.5), PTT (47 seconds), PT (35 seconds)	N-acetylcysteine 150 mg/kg over 15 minutes, 50 mg/kg over 4 hours, and then 100 mg/kg over 16 hours
Paracetamol overdose in a preterm neonate [1]	Isbister et al.	GA at birth: 29 weeks, weight: 2.200 kg, PMA at time of overdose: 37 + 0	136 mg/kg NG (×1)	121 mg/L 4 hours after last dose	No increase in LFT; increase in INR (1.3)	N-acetylcysteine 150 mg/kg over 15 minutes, 50 mg/kg over 4 hours, and then 100 mg/kg over 16 hours with activated charcoal
Lack of toxicity after paracetamol overdose in a extremely preterm neonate [2]	Porta et al.	GA at birth: 25 + 5 weeks, weight: 0.940 kg, PMA at time of overdose: 27 + 3	446 mg/kg IV (×1)	180 mg/L 5 hours after last dose	No increase in LFT; no increase in coagulation panel	N-acetylcysteine 150 mg/kg over 30 minutes, followed by 50 mL/kg over 4 hours, and then 100 mg/kg over 16 hours
Intravenous paracetamol overdose in a preterm infant during anesthesia [3]	Nevin and Shung	GA at birth: 28 + 0 weeks, weight: 2.600 kg, PMA at time of overdose: 35 + 0	146 mg/kg IV (×1)	117 mg/L 4 hours after last dose	No increase in LFT; increase in INR (1.26), PTT (41.8 seconds)	N-acetylcysteine 150 mg/kg over 30 minutes, followed by 50 mL/kg over 4 hours, and then 100 mg/kg over 16 hours; given vitamin K for 5 days
Medication error in an extremely low birth weight infant: paracetamol overdose [4]	Brener et al.	GA at birth: 27 + 0 weeks, weight: 0.750 kg, PMA at time of overdose: 28 + 2	226 mg/kg q 6 hours (×3) (route administration unclear)	480 mg/L (unknown timing after last dose)	No increase in LFT; no mention of coagulation panel	N-acetylcysteine 100 mg and then 50 mg until 17 doses received

## Discussion

There are several differences between preterm neonates and other patient populations that may account for apparent differences in their susceptibility to acetaminophen toxicity. Acetaminophen is metabolized mainly in the liver by sulfation, glucuronidation, and oxidation, with minimal amounts excreted unchanged in the urine. An oxidative pathway produces the toxic metabolite, N-acetyl-p-benzoquinone imine (NAPQI), which is conjugated with glutathione to non-toxic metabolites [5]. Glutathione levels after birth are higher in preterm neonates compared to term neonates. The common practice of administering parenteral nutrition soon after birth to preterm neonates provides amino acids that have been shown to counteract the rapid decline in glutathione concentrations that happens otherwise [6]. Pharmacokinetic models show that the enzymes involved in acetaminophen metabolism vary significantly with gestational age. These differences are most pronounced in extreme preterm neonates (23-25 weeks GA) when compared to adults: sulfation via sulfotransferase (SULT), 87.8%-91.9%; glucuronidation via UDP-glucuronosyltransferase (UGT), 3.51%-7.8% versus 48%; and oxidation via cytochrome P450 (CYP), 3.61%-3.78% versus 11% [5]. In older children and adults, elevated acetaminophen concentrations, in some cases, even therapeutic doses, can saturate the SULT and UGT pathways, thereby shifting metabolism toward the CYP pathway and resulting in increased NAPQI formation. The extent to which this saturation occurs in neonates is unknown [7].

However, there is no evidence of saturation, and concentrations of acetaminophen-cysteine and acetaminophen-mercapturate, markers for exposure to NAPQI, did not change appreciably across gestational ages (24-32 weeks) following a single 20 mg/kg/dose exposure to acetaminophen [8]. The lower fraction of oxidative metabolism, limited saturation of the sulfation pathway, and higher glutathione stores may all be protective of preterm neonates in acetaminophen overdose. In addition, despite a cumulative exposure of 200 mg/kg of intravenous acetaminophen, the serum concentration obtained 15 hours after the final dose was relatively low at 29.6 mg/L. This finding may partially reflect the unique body composition of extremely premature neonates, who possess a substantially higher percentage of total body water compared with older children and adults. The resulting expanded volume of distribution for hydrophilic medications such as acetaminophen may dilute peak serum concentrations despite significant total drug exposure.

N-acetylcysteine (NAC) is used to prevent or limit hepatotoxicity following acetaminophen overdose. Its use has been reported in the neonatal population; however, there is limited evidence for when to initiate NAC in neonates. The Rumack-Matthew nomogram is commonly used to predict the risk of hepatotoxicity and initiate NAC in adults following a single acute acetaminophen ingestion. This nomogram has not been validated in preterm neonates nor following multiple intravenous acetaminophen overdoses; hence, the Rumack-Matthew nomogram was not applied to this case.

Additionally, oral absorption of acetaminophen is by splanchnic circulation, resulting in 50% higher initial concentrations in the liver than observed following IV administration, which avoids the first-pass effect; thus, despite high serum concentrations, there is less drug in the liver to be converted to NAPQI initially [9]. Furthermore, serum acetaminophen concentration declines faster following IV overdose compared to enteral because of slower, ongoing absorption from the gastrointestinal tract, which could also potentially diminish the accuracy of the “treatment threshold” in predicting hepatotoxicity following IV overdose [9].

In our case, we were unable to accurately extrapolate the acetaminophen elimination half-life due to the intentional limitation of blood draws. With a 26 + 6 week infant, weighing 640 g, an estimated total blood volume of approximately 58-64 mL (90-100 mL/kg), along with an abnormal coagulation profile prior to NAC administration, further phlebotomy posed a risk of iatrogenic harm. As a result, only two acetaminophen concentrations were obtained: one at the time the overdose was recognized and a second level that was undetectable 10 hours after NAC was initiated. While half-life estimation might have provided additional pharmacokinetic insight and supported treatment rationale, the infant’s clinical instability and the need to minimize blood loss prevented additional sampling.

In our case, the transient elevation in coagulation parameters occurred in the absence of biochemical evidence of hepatocellular injury. Acetaminophen has been associated with isolated prolongation of coagulation studies through effects on vitamin K-dependent clotting factor activity independent of hepatic necrosis. Alternatively, the infant’s underlying spontaneous intestinal perforation and critical illness may have contributed to the observed coagulopathy. The etiology of this laboratory abnormality remains uncertain; however, these findings suggest that coagulation abnormalities may occur following acetaminophen overdose in premature neonates even when liver function tests remain normal. The profound but transient elevation in INR despite normal transaminases represents a notable clinical paradox. Acetaminophen has been reported to cause isolated prolongation of coagulation parameters through interference with vitamin K-dependent clotting factor activity independent of overt hepatocellular necrosis. Alternatively, the infant’s concurrent spontaneous intestinal perforation and critical illness may also have contributed to a localized consumptive coagulopathy. Clinicians should recognize that normal hepatic transaminases following neonatal acetaminophen overdose do not necessarily exclude significant systemic or extrahepatic coagulation abnormalities.

This case highlights the complex interplay of developmental pharmacokinetics and clinical decision-making in the management of acetaminophen overdose in premature neonates. While pharmacologic and metabolic differences, such as higher reliance on sulfation, reduced oxidative metabolism, and elevated glutathione levels, may confer some protection against hepatotoxicity, the limited and heterogeneous data in this population underscore the need for caution. As the use of acetaminophen in NICUs has expanded in the treatment of patent ductus arteriosus, understanding the pharmacokinetics, metabolism, and toxicity thresholds in premature neonates has become increasingly important, as well as the treatment of acetaminophen overdose. Until more robust data become available, clinicians must rely on a combination of pharmacologic principles, clinical judgment, and a low threshold for treatment when managing acetaminophen overdose in premature infants.

This case also illustrates a series of patient safety failures consistent with the Swiss cheese model, in which multiple latent system gaps aligned to permit the overdose to reach the patient: the acetaminophen preparation was non-formulary; its entry into the electronic medical record (EMR) lacked automated overdose warnings; and the dosing error passed through several layers of clinical oversight, including the ordering physicians, NICU pharmacy verification, and bedside nursing checks. As part of a system-wide safety response, and coinciding with the transition to a new EMR platform, standardized IV acetaminophen dosing recommendations and maximum dose alerts have now been embedded into electronic order sets to reduce the risk of recurrence.

In our case, it remains unclear why the infant experienced a significant and transient alteration in coagulation profile despite normal liver function testing, highlighting potential gaps in our understanding of acetaminophen’s extrahepatic effects or early signs of hepatic stress in this population. One limitation of this case was the restricted number of laboratory assessments due to intentional minimization of blood draws to prevent iatrogenic blood loss in this extremely low-birth-weight infant. The infant had no active signs of bleeding, and routine head ultrasounds were stable, monitoring an already existing bilateral grade 4 intraventricular hemorrhage. This case contributes to the growing but still limited literature and emphasizes the urgent need for population-specific guidelines in this vulnerable group.

## Conclusions

Extremely premature infants may be less susceptible to acetaminophen-induced hepatotoxicity than older children and adults, potentially due to developmental differences in drug metabolism, including reduced CYP450-mediated oxidative metabolism and greater reliance on sulfation pathways. However, the transient coagulation abnormalities observed in this case, despite normal liver function tests, highlight the possibility that acetaminophen toxicity in premature neonates may manifest differently than in other populations. As acetaminophen use continues to expand in neonatal intensive care units, particularly for patent ductus arteriosus closure in extremely low-birth-weight infants, a better understanding of the unique pharmacokinetic, toxicokinetic, and clinical effects of overdose is needed. Continued case reporting and dedicated pharmacologic studies are essential to establish evidence-based toxicity thresholds, guide N-acetylcysteine use, and optimize the management of acetaminophen overdose in this vulnerable population.

## Appendices

Supplemental information on search criteria for literature review

PubMed

("Infant, Premature"[Mesh] OR "premature infant*"[tiab] OR "preemie*"[tiab] OR "micro preemie*"[tiab] OR "micropreemie*"[tiab] OR "preterm infant*"[tiab] OR "premature neonate*"[tiab] OR "preterm neonate"[tiab] OR "premature newborn*"[tiab] OR "preterm newborn"[tiab]) AND ("Acetaminophen"[Mesh] OR "acetaminophen"[tiab] OR "APAP"[tiab] OR "Tylenol"[tiab] OR "N-Acetyl-p-aminophenol"[tiab] OR "paracetamol"[tiab]) AND ("Drug Overdose"[Mesh:NoExp] OR "overdos*"[tiab])

EMBASE

('prematurity'/exp OR 'premature infant*':ti,ab OR 'preemie*':ti,ab OR 'micro preemie*':ti,ab OR 'micropreemie*':ti,ab OR 'preterm infant*':ti,ab OR 'premature neonate*':ti,ab OR 'preterm neonate':ti,ab OR 'premature newborn*':ti,ab OR 'preterm newborn':ti,ab) AND ('paracetamol'/exp OR 'acetaminophen':ti,ab OR 'apap':ti,ab OR 'tylenol':ti,ab OR 'n-acetyl-p-aminophenol':ti,ab OR 'paracetamol':ti,ab) OR ('intoxication'/exp OR 'drug overdose'/de OR 'drug intoxication'/de OR "overdos*":ti,ab) AND ('article'/it OR 'review'/it)

CINAHL

((MH "Infant, Premature") OR TI("premature infant*" OR "preemie*" OR "micro preemie*" OR "micropreemie*" OR "preterm infant*" OR "premature neonate*" OR "preterm neonate" OR "premature newborn*" OR "preterm newborn") OR AB("premature infant*" OR "preemie*" OR "micro preemie*" OR "micropreemie*" OR "preterm infant*" OR "premature neonate*" OR "preterm neonate" OR "premature newborn*" OR "preterm newborn")) AND ((MH "Acetaminophen") OR TI("acetaminophen" OR "APAP" OR "Tylenol" OR "N-Acetyl-p-aminophenol" OR "paracetamol") OR AB("acetaminophen" OR "APAP" OR "Tylenol" OR "N-Acetyl-p-aminophenol" OR "paracetamol")) AND (MH "Overdose") OR (TI(overdos*) OR AB(overdos*))

Web of Science

(TS=("premature infant*" OR "preemie*" OR "micro preemie*" OR "micropreemie*" OR "preterm infant*" OR "premature neonate*" OR "preterm neonate" OR "premature newborn*" OR "preterm newborn") AND TS=("acetaminophen" OR "APAP" OR "Tylenol" OR "N-Acetyl-p-aminophenol" OR "paracetamol") AND TS=(overdos*)) AND (DT==("REVIEW" OR "ARTICLE"))

CINAHL

((MH "Overdose") OR (TI(overdos*) OR AB(overdos*))) AND (((MH "Infant, Premature") OR TI("premature infant*" OR "preemie*" OR "micro preemie*" OR "micropreemie*" OR "preterm infant*" OR "premature neonate*" OR "preterm neonate" OR "premature newborn*" OR "preterm newborn") OR AB("premature infant*" OR "preemie*" OR "micro preemie*" OR "micropreemie*" OR "preterm infant*" OR "premature neonate*" OR "preterm neonate" OR "premature newborn*" OR "preterm newborn")) AND ((MH "Acetaminophen") OR TI("acetaminophen" OR "APAP" OR "Tylenol" OR "N-Acetyl-p-aminophenol" OR "paracetamol") OR AB("acetaminophen" OR "APAP" OR "Tylenol" OR "N-Acetyl-p-aminophenol" OR "paracetamol")) AND ((MH "Overdose") OR (TI(overdos*) OR AB(overdos*))))
